# Treatment of Spider Phobia Using Repeated Exposures and Adjunctive Repetitive Transcranial Magnetic Stimulation: A Proof-of-Concept Study

**DOI:** 10.3389/fpsyt.2022.823158

**Published:** 2022-03-15

**Authors:** Michael K. Leuchter, Benjamin M. Rosenberg, Giuditta Schapira, Nicole R. Wong, Andrew F. Leuchter, Anastasia L. McGlade, David E. Krantz, Nathaniel D. Ginder, Jonathan C. Lee, Scott A. Wilke, Reza Tadayonnejad, Jennifer Levitt, Katharine G. Marder, Michelle G. Craske, Marco Iacoboni

**Affiliations:** ^1^TMS Clinical and Research Program, Neuromodulation Division, Semel Institute for Neuroscience and Human Behavior at UCLA, Los Angeles, CA, United States; ^2^Department of Psychiatry and Biobehavioral Sciences, David Geffen School of Medicine at UCLA, Los Angeles, CA, United States; ^3^Department of Psychology, University of California, Los Angeles, Los Angeles, CA, United States; ^4^David Geffen School of Medicine at UCLA, Los Angeles, CA, United States; ^5^Division of the Humanities and Social Sciences, California Institute of Technology, Pasadena, CA, United States

**Keywords:** intermittent theta-burst stimulation (iTBS), phobia, spider phobia, neuromodulation, transcranial magnetic stimulation (TMS), repetitive transcranial magnetic stimulation (rTMS), behavioral

## Abstract

**Background:**

Specific phobias represent the largest category of anxiety disorders. Previous work demonstrated that stimulating the ventromedial prefrontal cortex (vmPFC) with repetitive Transcranial Magnetic Stimulation (rTMS) may improve response to exposure therapy for acrophobia.

**Objective:**

To examine feasibility of accelerating extinction learning in subjects with spider phobia using intermittent Theta Burst Stimulation (iTBS) rTMS of vmPFC.

**Methods:**

In total, 17 subjects with spider phobia determined by spider phobia questionnaires [Spider Phobia Questionnaire (SPQ) and Fear of Spiders questionnaire (FSQ)] underwent ratings of fear of spiders as well as behavioral and skin conductance data during a behavioral avoidance test (BAT). Subjects then received a sequential protocol of *in vivo* spider exposure followed by iTBS for three sessions administered to either active or control treatment sites (vmPFC [*n* = 8] or vertex [*n* = 9], respectively), followed 1 week later by repetition of questionnaires and BAT.

**Results:**

All subjects improved significantly regardless of group across both questionnaires (FSQ η^2^ = 0.43, *p* = 0.004; SPQ η^2^ = 0.39, *p* = 0.008) and skin conductance levels during BAT (Wald χ^2^ = 30.9, *p* < 0.001). Subjects in the vmPFC group tolerated lower treatment intensity than in the control group, and there was a significant correlation between treatment intensity, BAT subjective distress improvement, and physiologic measures (all ρ > 0.5).

**Conclusion:**

This proof-of-concept study provides preliminary evidence that a sequential exposure and iTBS over vmPFC is feasible and may have rTMS intensity-dependent effects on treatment outcomes, providing evidence for future areas of study in the use of rTMS for phobias.

## Introduction

Specific phobias represent the largest category of anxiety disorders in the world with an estimated lifetime prevalence of 7.4–15% (1–3). The core diagnostic criterion is persistent fear, anxiety, or avoidance of a specific stimulus that results in a clinically significant impairment in daily functioning (3, 4).

Exposure therapy (ET) is currently considered the gold-standard treatment for specific phobia, with an estimated 75% of patients seeing clinical benefit compared with placebo (5) ET extinguishes conditional fear through repeated, unreinforced exposure to the feared stimulus repeatedly over time (6, 7). 25–30% of patients will not respond to ET, however, and many phobic patients avoid or refuse treatment or discontinue treatment before completion (4, 5, 8, 9).

Repetitive Transcranial Magnetic Stimulation (rTMS) is a novel treatment utilized for Major Depressive Disorder (MDD) (10–12), obsessive-compulsive disorder (OCD) (13, 14), substance dependence (15–17), and other neuropsychiatric disorders (18–20). rTMS uses low-intensity electromagnetic energy to stimulate critical hubs of brain networks and “reset” function (21). rTMS stimulation of a particular target is believed to alter connectivity between the target and other brain regions in the same network (22–25). Studies suggest that rTMS may be efficacious in the treatment of anxiety disorders (14, 26–29), consistent with evidence showing that stimulation of the medial prefrontal cortex modulates discrimination of learned safety and threat cues (30) and may ameliorate symptoms in specific phobia (26, 30–33).

The ventromedial prefrontal cortex (vmPFC) is a promising rTMS treatment target (34, 35) that is thought to play a central role in the etiology, maintenance, and treatment of anxiety disorders due to its functional and structural connectivity with subcortical regions involved in fear learning and recall, such as the amygdala (36–41) and hippocampus (41–44). One study found that individuals with acrophobia who received rTMS to the vmPFC along with ET demonstrated a greater improvement compared to those receiving ET and sham stimulation (35).

In this proof-of-concept pilot study, we aimed to examine the feasibility and potential benefit of using rTMS stimulation of vmPFC to augment the efficacy of ET for the treatment of spider phobia. Previous work examining the use of rTMS in spider phobia has examined the immediate effects of a single session of rhythmic rTMS (32, 33). We utilized multiple sessions of intermittent Theta-Burst Stimulation (iTBS, which has recently been shown to have efficacy similar to rhythmic rTMS while reducing treatment time by as much as 90%) and examined durability of effects 1 week after treatment (45–48). The protocol in this study is similar to later work in acrophobia that demonstrated positive results (35). We hypothesized that iTBS stimulation of vmPFC would be well tolerated in subjects with spider phobia and that those receiving iTBS vmPFC stimulation prior to a behavioral avoidance test (BAT) would exhibit greater willingness to approach a novel spider, lower self-reported distress during the BAT, and greater reductions in skin conductance level (SCL, a physiologic measure commonly used in studies of phobias, including spider phobia) (33, 49–51) compared to those receiving control stimulation. Additional exploratory analyses assessed the extent to which stimulation intensity to the vmPFC was associated with these same dependent variables.

## Materials and Methods

### Design

Twenty-two rTMS-naïve subjects meeting criteria for spider phobia were recruited and randomized 1:1 to treatment with iTBS stimulation of vmPFC (active) or Cz (control) following each of three repeated exposures to live spiders.

**FIGURE 1:**
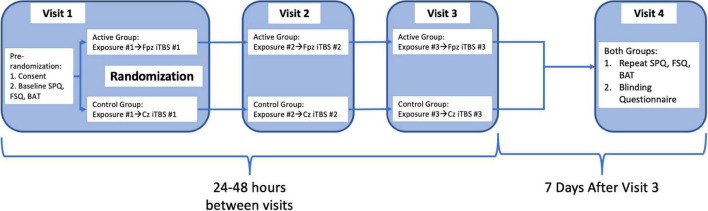
Graphical representation of procedural flow of study steps as detailed in the section “Procedures.”

Subjects and research staff conducting the behavioral assessments and exposures were blinded to rTMS treatment condition. After initial screening, subjects completed online screening for eligibility and gave written informed consent in accordance with the Declaration of Helsinki (52). The ClinicalTrials.gov Identifier is NCT04019054. After informed consent, subjects completed baseline Spider Phobia Questionnaire (SPQ) and Fear of Spiders questionnaire (FSQ), and a behavioral avoidance test (BAT), followed by an exposure session and iTBS administered to either an active or control stimulation site based on group assignment (randomly assigned by non-rater study staff on enrollment prior to iTBS treatment). Two subsequent exposure plus iTBS treatments were administered at 24–48 h intervals (49, 51, 53, 54). At a final visit 1 week later, the SPQ and FSQ were repeated followed by an identical BAT to that administered at the initial visit. Both subjects and raters then completed a blinding questionnaire asking them to state whether they were assigned to active or control group. Subjects were debriefed by the principal investigator on completion of their participation, and all subjects completed participation within 2 weeks. Full timeline also outlined in Figure 1.

### Subjects

Inclusion criteria were: at least 18 years of age, English-speaking, and a pre-screening SPQ score of at least 18. Exclusion criteria were: inability to provide informed consent, presence of suicidal ideation, history of a diagnosed mood, psychotic, or anxiety disorder (other than specific phobia), active prescriptions for medications known to affect seizure threshold, elevated anxiety and depression screening tool scores (PHQ9 > 10, GAD7 > 10, HAMD > 8 or suicide item score >2), history of neuromodulation treatments for any condition (rTMS, direct current stimulation, electroconvulsive therapy, or vagus nerve stimulation), history of a condition affecting the central nervous system (stroke, seizure, neurocognitive disorders, and intracranial implants), current pregnancy, increased seizure risk due to medication or family history, and known bee or insect allergies.

### Procedures

#### Behavioral Avoidance Testing

A behavioral avoidance testing (BAT) with nine standardized steps was utilized as previously described (administered by research staff blinded to subject group assignment) (49, 51). Prior to initiation of the BAT, baseline skin conductance level (SCL) was recorded for 2 min from two electrodes attached to the second and third fingers of the subjects’ non-dominant hand. SCL was continuously measured with BIOPAC MP150 hardware and AcqKnowledge version 4.2 software (BIOPAC Systems, Inc., Goleta, CA, United states). Subjects were exposed to one of two Chilean Rose Tarantulas (*Grammostola rosea*) throughout the BAT. Subjects were instructed to approach the spider as closely as possible according to a series of nine standardized sequential steps lasting 30 s each. The first step involved standing 5 feet away from the spider, and the last step involved the subject touching the spider’s back leg continuously with the tip of their index finger. When subjects failed to complete a step in the sequence (such as withdrawing during or before completion of a step), the BAT was terminated and the number of steps fully completed was recorded. Prior to the BAT, subjects rated their overall confidence and distress about completing all nine steps. During the BAT, they rated their confidence and anticipatory distress prior to each step, and maximum distress after each step, on a scale from 0 to 100.

#### *In vivo* Exposures

*In vivo* exposure was conducted with a different Chilean Rose Tarantula from the one used in the BAT immediately prior to each of the three iTBS treatments. Exposures consisted of 10 identical exposure trials (involving subjects hovering their ungloved hand 3 in. above the tarantula in its terrarium) of 30-s duration with a 30-s pause between trials, for a total task duration of 10 min.

#### Transcranial Magnetic Stimulation

All rTMS treatments were performed with either the Magstim Rapid 2 stimulator using a 70 mm butterfly coil (Magstim, Whitland, NSW, United Kingdom) or the Magventure MagPro R30 stimulator using a 75 mm butterfly coil (MagVenture, Farum, Denmark). Resting motor threshold (RMT) was determined for all subjects as the minimum stimulus intensity necessary to elicit a motor response in the right abductor pollicis brevis or first dorsal interosseus muscles for ≥50% of stimuli applied to the motor cortex (55). RMT, a stable intra-subject parameter, was performed before the repeated exposures on the first day to minimize time delay from repeated exposures to TMS (56–58). Each participant used only one coil type (i.e., their MT was measured on one device and they were treated with that device for all three sessions), and coil assignment was based on device availability in the UCLA TMS Clinical and Research Service. All subjects, regardless of group assignment, then were instructed to hold an ice pack on their forehead (over the vmPFC stimulation site) for 5–7 min before beginning rTMS. This was done primarily to minimize discomfort for the active group, though it was done for all subjects in order to maintain blinding for subjects and raters. To maintain blinding, raters did not observe rTMS treatments, and subjects were not informed of the difference between groups during their participation in the study.

**FIGURE 2:**
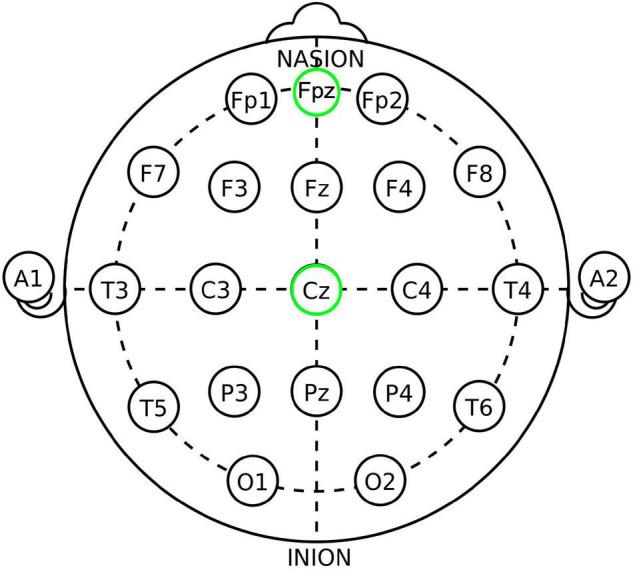
The 10–20 International system of EEG electrode placement, from Wikipedia.org “10–20 system (EEG)” and modified. Fpz (placement utilized in this study for active/vmPFC placement) and Cz (placement utilized for control/Cz placement) locations circled in green.

Subjects then underwent the iTBS protocol. For the active group, the TMS coil was positioned over the subject’s vmPFC (as determined using position Fpz, or the nasion, of the international 10–20 EEG electrode system) as seen in Figure 2 using a modified version of the Beam F3 method (30, 31, 35, 59–61). Stimulation was delivered at 100% of the subject’s RMT (using a ramp-up protocol starting from 80% RMT and advancing as tolerated) in bursts of three pulses at a frequency of 50 Hz every 200 ms on top of a 5 Hz carrier wave. Pulse delivery occurred over 2 s and was repeated every 10 s, 20 times in succession, for a total of 600 pulses delivered in 192 s (31, 35, 45, 60).

Intermittent theta burst stimulation in the control group consisted of the same stimulation parameters applied to the vertex (as determined using position Cz, of the International 10–20 EEG Electrode System) in place of vmPFC. The Cz placement was chosen for control because: (1) it is a commonly used control site in TMS studies and has a similar risk profile to other TMS sites (23, 62–67), (2) its position over the vertex minimizes the amount of cortex stimulated (67), (3) the stimulated area is not known to be associated with the circuitry examined in this experiment (32, 35, 39, 67, 68), and (4) it is generally associated with low rates of scalp discomfort and no clinically observed behavioral or mood effects (64, 66, 67, 69). The difference between the active and control groups was not disclosed to subjects until after completion of their participation.

### Measures

The first outcome measures examined were fear of spiders as determined using both the 31-item spider phobia questionnaire (SPQ) and the 18-item fear of spiders questionnaire (FSQ) (53, 54).

During each BAT, skin conductance level (SCL) served as a physiological marker of fearful arousal. Three SCL values were calculated: (1) Baseline SCL, i.e., mean SCL during a 2-min period prior to initiation of each BAT, (2) Anticipatory SCL, i.e., mean SCL during a 1-min anticipation period after reading the subject a description of the BAT but prior to BAT initiation, and (3) mean SCL during each fully completed BAT step (51). Data were filtered using a finite impulse response (FIR) low pass filter with the frequency cutoff fixed at 2 Hz; no significant movement confounds emerged during data extraction.

In addition to SCL, confidence and distress rating were obtained using a visual analog rating scale ranging from 0 to 100 (0 = no distress/no confidence; 100 = severe distress/complete confidence). Anticipatory distress (AntD) and confidence ratings were obtained before beginning the BAT and prior to beginning each step, and maximum distress ratings (MaxD) were obtained after each step. The number of BAT steps fully completed (0–9) served as a behavioral measure of avoidance related to fear of spiders.

Variable experiment parameters recorded included post-rTMS pain scores as rated by a brief McGill questionnaire, minute delay from repeated exposures to initiation of rTMS treatment, maximum rTMS treatment intensity tolerated, and any early treatment terminations due to rTMS tolerability (70). Baseline demographic and psychiatric screening information was gathered but not included in statistical analysis.

### Statistical Analysis

The number of statistical tests performed was minimized and multi-level mixed-effects models (MLMs) and non-parametric tests were utilized as appropriate based on the sample size and non-normal distributions of multiple outcome measures. Group assignment, timepoint (pre or post-treatment), and BAT step were examined in association with each measure acquired during the BATs (anticipatory distress, maximum distress, SCL at each step) using MLMs in Stata version 16. All other analyses were performed in SPSS (version 26.0.0.0). Demographics and experimental parameters were analyzed for between-group differences using 2-sided Mann–Whitney U Tests. Changes in self-reported fear of spiders (SPQ and FSQ scores) were analyzed using a repeated-measures MANOVA (Greenhouse Geisser correction was applied in case of non-sphericity). Chi-square analyses were performed to validate blinding of the study using the previously described blinding questionnaires. A *p*-value of <0.05 was considered statistically significant for all tests.

## Results

### Participants—Demographics, Baseline, and Experimental Parameters

**FIGURE 3:**
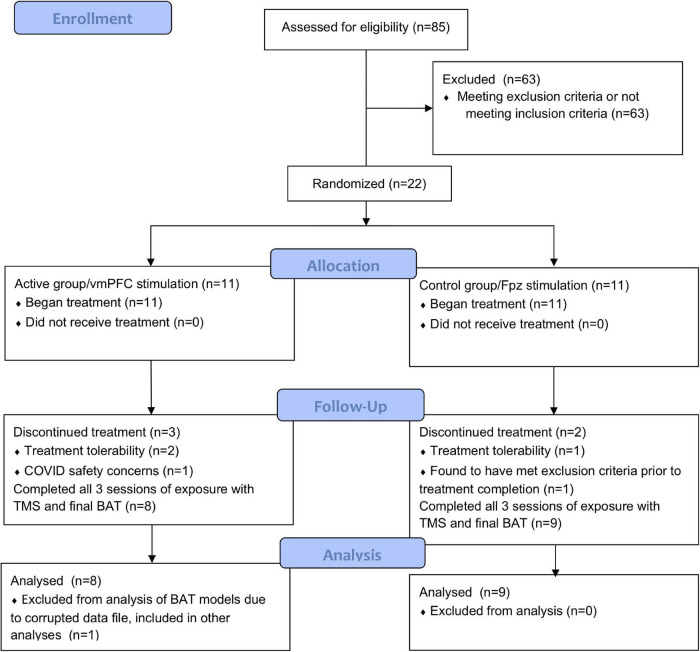
CONSORT flow diagram of the described study.

In total, 85 subjects were screened and 22 enrolled in the study. Gender, age, and screening survey data are shown in Table 1. Two subjects withdrew (one from each group) after the first rTMS session for tolerability reasons as did one additional subject from the active group during the third rTMS session. One subject from the control group with a history of MDD and GAD was excluded by staff prior to completion of the study due to meeting exclusion criteria. One subject from the active group withdrew after the first rTMS session due to safety concerns related to COVID-19 in March of 2020. No subjects had active prescriptions for antidepressant or anxiolytic medications at the time of the study. Subject enrollment and completion is further shown in our CONSORT diagram (Figure 3). Test steps completed by participants in each group at pre- and post-treatment are shown in Table 2.

**TABLE 1:** Baseline demographic and screening data, as well as pre-treatment baseline measurement of outcome variables and experimental parameters.

	Active (*N* = 8)		Control (*N* = 9)	
Demographics and Screening	M	SEM	M	SEM
Female Subjects	7		4	
Male Subjects	1		5	
Age	21.1	2.3	21.1	3.8
PHQ9 Score^a^	1.9	1.6	1.9	1.9
GAD7 Score^b^	3.4	2.9	2.2	2.1
HAMD Score^c^	2.4	2.3	2.1	2.1
**Subjective Questionnaires**				
SPQ^d^	21.6	4.0	22.7	3.8
FSQ^e^	93.9	16.0	96.9	9.4
**BAT Behavioral^f^**				
Steps	7.3	1.3	6.4	1.5
*Anticipation Step* ^g^				
Distress	61.9	24.0	62.2	22.0
Confidence	45.9	32.0	62.8	21.0
*Final Step* ^h^				
Anticipatory Distress	66.9	28.0	69.4	24.0
Maximum Distress	65.9	28.0	71.1	21.0
**BAT Physiologic/SCL^i^**				
Baseline^†^	2.91	2.70	7.99	7.30
Anticipation Step^g^	4.63	4.20	10.70	8.30
Final Step^h^	7.19	5.90	12.20	9.60
**Treatment Parameters**				
*Exposures*				
Number completed	9.5	0.9	9.3	1.1
*TMS*				
Time delay^k^	16.3	4.1	14.4	3.6
Intensity Tolerated^l^*	90.8	8.1	98.8	2.6
Post-TMS Pain^m^	3.3	4.2	1.7	1.9

Statistical testing for baseline comparison performed only on average treatment intensity tolerated and baseline skin conductance level (Mann–Whitney U Test). * indicates significance at p < 0.05 level, ^†^indicates no statistical significance. Other variables were not compared between groups. ^a^PHQ9 score, maximum 27, ^b^GAD7 score, maximum 21, ^c^HAMD score, maximum 52, ^d^Spider Phobia Questionnaire (SPQ) score, maximum 31, ^e^Fear of Spiders Questionnaire (FSQ) score, maximum 126, ^f^Behavioral measures examined during Behavioral Avoidance Test (BAT), ^g^Measures examined during anticipation step of BAT, ^h^Measures examined during the last step each individual subject completed during both pre and post-treatment BATs, referred to as the final step or final common step, ^i^Physiologic measure, skin conductance level (SCL) as examined during the BAT, ^k^Minute delay from the end of the exposure treatment to the initiation of TMS treatment, ^l^Maximum intensity tolerated by subjects during TMS (as% of motor threshold), ^m^Post-TMS discomfort experienced by subjects as rated on the short-form McGill scale, maximum 45.

**TABLE 2:** Number of participants who completed each BAT step before and after treatment in each group.

	Active (*N* = 8)		Control (*N* = 9)	
Step	Pre	Post	Pre	Post
1	8	8	9	9
2	8	8	9	9
3	8	8	9	9
4	8	8	9	8
5	8	7	8	7
6	7	7	7	7
7	6	6	4	6
8	4	5	2	6
9	1	1	1	4

*No statistically significant between or within-group differences by MLM examining contributions of timepoint and group to steps completed.*

**FIGURE 4:**
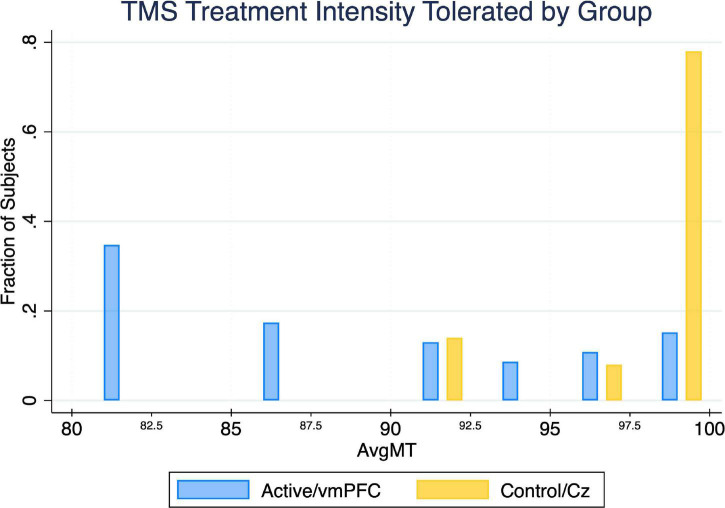
Between-group comparison of average TMS treatment intensity tolerated (noted as a percent of the resting motor threshold). Utilizing a 2-sided Mann–Whitney U Test, we found the maximum TMS intensity tolerated by the active treatment group is significantly lower (*p* = 0.027) than the control treatment group by an average of 8.0%.

The active treatment group tolerated significantly less intense iTBS stimulation than the control group (*U* = 13.5, *p* < 0.05) (Table 1). The majority of subjects in the control group tolerated 100% treatment intensity for the entire duration of treatment, whereas less than 40% of the active group was able to do so (Figure 4).

### Self-Reported Survey Outcomes

**FIGURE 5:**
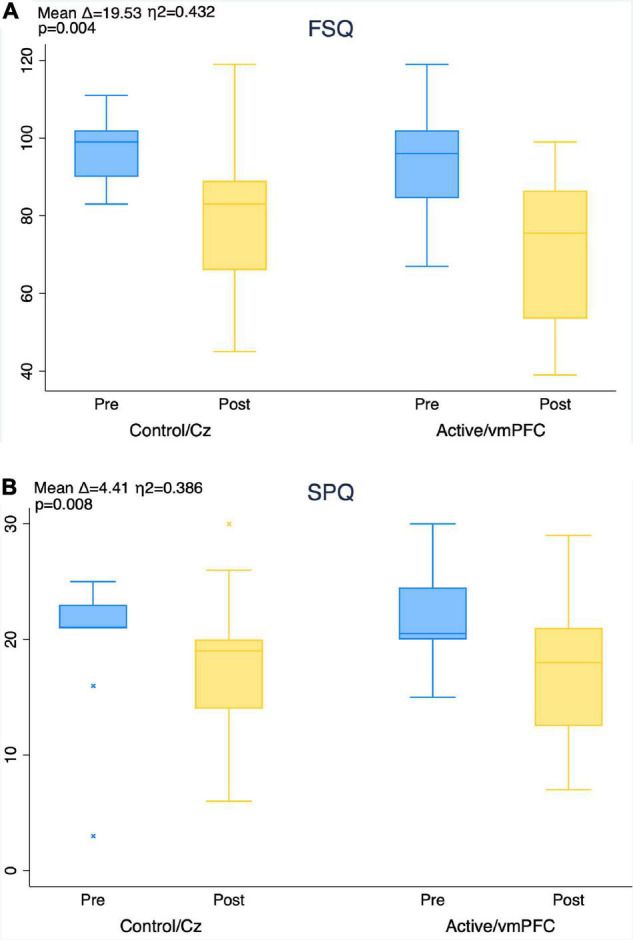
Improvement in fear of spiders questionnaire (FSQ, **A**) and spider phobia questionnaire (SPQ, **B**) scores from pre- to post-treatment by group. Mean score improvement (mean delta), MANOVA level of significance, and effect size (eta-squared) of aggregate subject pool provided for timepoint comparison in each figure. No between-group effects found using MANOVA.

There were significant decreases in subjective distress as indicated by both the FSQ and SPQ for both groups over time with no significant effect of treatment group (FSQ η^2^ = 0.43, *p* = 0.004; SPQ η^2^ = 0.39, *p* = 0.008) (Figures 5A,B).

### Multi-Level Models, Physiological, and Observed Behavioral Outcomes

Three-way effect models showed significant effects for group, BAT step, and time (Wald χ^2^: χ^2^ SCL = 51.9, χ^2^ AntD = 167.8, χ^2^ MaxD = 105.3; all *p* < 0.001) with no significant interactions for any of the outcome measures (SCL: β = −0.002, SEM = 0.06, *p* = 0.97; AntD: β = −0.15, SEM = 0.13, *p* = 0.25; MaxD: β = −0.001, SEM = 0.14, *p* = 1.0).

**FIGURE 6:**
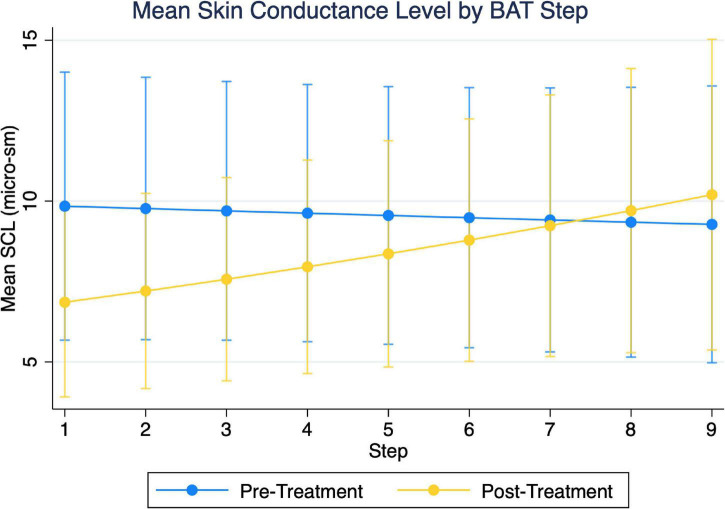
Depiction of two-variable multi-level models of skin conductance level (SCL), as a function of step and timepoint. Initial three-variable model including group were found not to have significant three-way interaction.

Examining two-way group and timepoint effect models of SCL, we found the overall model to be significant (χ^2^ = 47.9, *p* < 0.001) in addition to a significant group-x-timepoint interaction (β = 0.50, SEM = 0.108, *p* < 0.01). Simple effects indicated this was due to group differences during the pre-treatment BAT, not due to differences with treatment, and therefore not further examined. Similarly, examining the two-way timepoint and step models, we found the overall model to be significant (χ^2^ = 30.90, *p* < 0.001) in addition to a significant timepoint-x-step interaction (β = 0.06, SEM = 0.027, *p* = 0.029). Mean SCL decreased with active treatment up to and including step 4 of the BAT (Figure 6 and Table 3). There appeared to be a crossover effect at step 7 (Figure 4), although examination of this possible effect is complicated by the diminished number of subjects who completed more than 6 steps before and after treatment.

**TABLE 3:** Coefficient, standard error of the mean, and level of significance of timepoint difference for skin conductance multi-level model.

Step	β	SEM	*p*
1**	−2.99	0.95	0.002
2**	−2.57	0.78	0.001
3**	−2.13	0.64	0.001
4**	−1.67	0.58	0.004
5	−1.19	0.63	0.06
6	−0.70	0.77	0.37
7	−0.18	0.99	0.86
8	0.36	1.24	0.77
9	0.92	1.53	0.55

**Indicates significance at the p < 0.01 level.

Examining AntD, we again found the overall model with regard to group and timepoint to be significant (χ^2^ = 158.7, *p* < 0.001), as well as a significant group-x-timepoint interaction (β = 1.27, SEM = 0.26, *p* < 0.001) as well as the model of group and step and their interaction effects (*p* < 0.001, χ^2^ = 121.8; β = −0.15, SEM = 0.075, *p* = 0.047). Simple effects again indicated these were due to group differences during the pre-treatment BAT, not due to differences with treatment, and therefore not further examined. We additionally found that the model of step and timepoint was significant (χ^2^ = 114.0, *p* < 0.001) with no significant step-x-timepoint interaction (β = −0.03, SEM = 0.06, *p* = 0.63).

For MaxD, the model of group and timepoint was significant (χ^2^ = 96.9, *p* < 0.001) with a significant group-x-timepoint interaction (β = 1.31, SEM = 0.30, *p* < 0.001). Similarly, the model of group and step and their interaction effects were found to be significant (χ^2^ = 78.44, *p* < 0.001; β = −0.19, SEM = 0.082, *p* = 0.024). Simple effects again indicated these were due to group differences during the pre-treatment BAT, not due to differences with treatment, and therefore not further examined.

### *Post-hoc* Exploratory Analyses: Treatment Intensity Relationships

**FIGURE 7:**
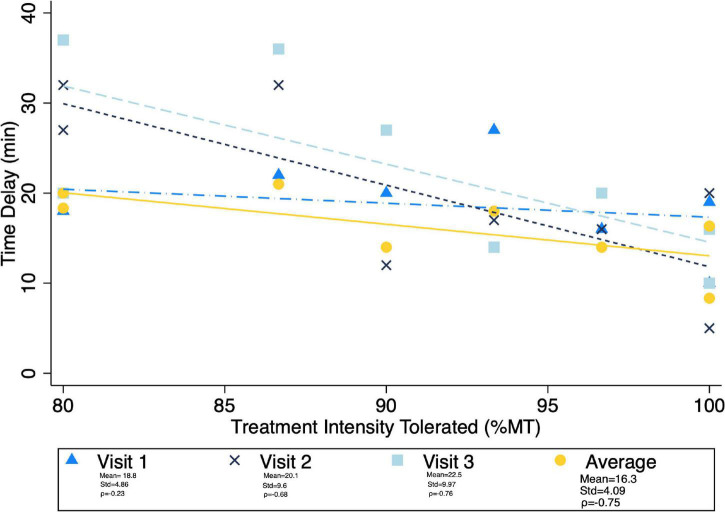
Correlation between average maximum treatment intensity tolerated (noted as a percent of resting motor threshold) and time delay from exposure to TMS. Mean time delay, standard deviation of the delay, and spearman correlation coefficient between treatment intensity and time delay noted for each visit. Pearson correlation was notably lowest at visit 1, and ρ > = 0.7 for all other visits and average delay.

**FIGURE 8:**
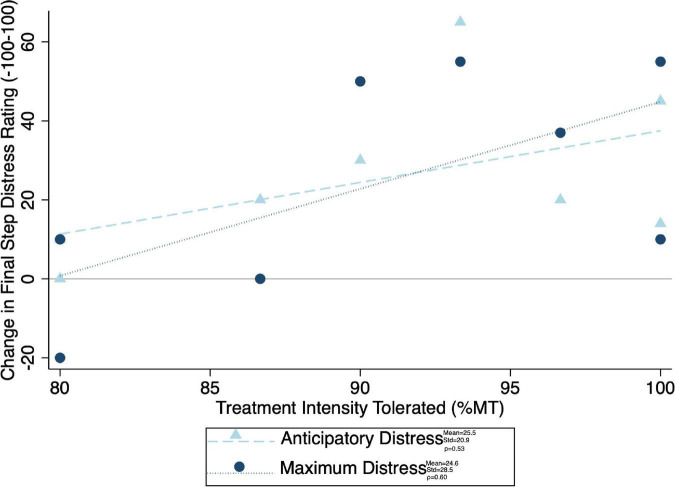
Correlation between maximum treatment intensity tolerated and final common step distress (both anticipatory and maximum experienced distress shown). Change in distress was calculated as pre minus post, with positive values indicating a decrease in distress with treatment. Mean change in distress, standard of deviation of the change in distress, and spearman correlation coefficient (ρ) for both anticipatory and maximum distress shown.

**FIGURE 9:**
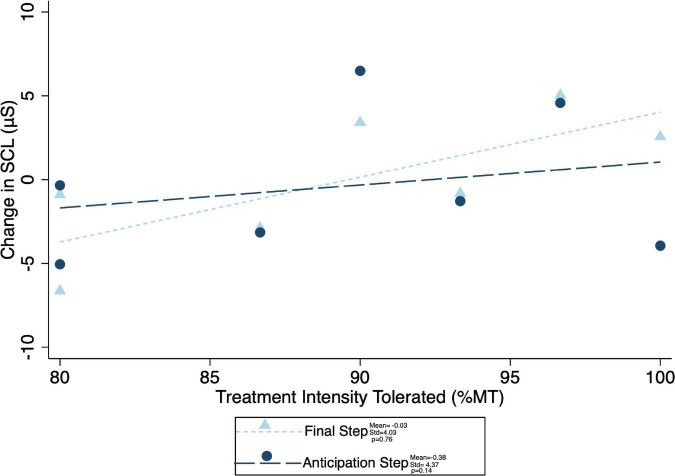
Correlation between maximum treatment intensity tolerated and both anticipation step and mean final common step skin conductance level change (difference in mean skin conductance during the final step that was completed during both the pre- and post-treatment BATs). Change in SCL was calculated as pre minus post, with positive values indicating a decrease in SCL with treatment. Mean change in SCL, standard of deviation of the SCL change, and spearman correlation coefficient (ρ) for both anticipation and final common steps shown.

Given the significant differences in treatment intensity by group (Figure 4), a *post-hoc* analysis was performed to examine the within-group correlation between treatment intensity and (1) other experimental parameters, as well as (2) outcome measures of interest. There was a significant correlation between treatment intensity and length of delay (mean delay = 15.25 min, SD = 3.84 min) from the end of the exposures to the initiation of TMS on average (ρ = 0.75) and for the latter two of the three visits (Visit 1 ρ = 0.23, visit 2 ρ = 0.68, visit 3 ρ = 0.75) (Figure 7). Individuals who tolerated greater treatment intensity also experienced a greater decrease in subjective distress from pre- to post- treatment BATs (AntD ρ = 0.53, MaxD ρ = 0.60; Figure 8). A similar relationship was observed between treatment intensity and changes in mean SCL during the final BAT step each subject completed both before and after treatment (ρ = 0.76; Figure 9).

### Validation of Blinding

Blinding validation study utilizing chi-square tests comparing correct and incorrect group assignment guesses by subjects and raters did not significantly deviate from random chance (Table 4).

**TABLE 4:** Number of subjects and raters correctly guessing group assignment after treatment and repeat BAT.

	Active (*N* = 8)		Control (*N* = 9)	
	Correct	Incorrect	Correct	Incorrect
Subject	5	3	4	5
Rater	4	4	5	4

*Two chi-square analyses were performed, one for raters and one for subjects, and no deviation from 50/50 chance with regarding to determination of group assignment was found (p = 0.45 subjects, p = 0.82 raters).*

## Discussion

These findings support the feasibility and potential efficacy of rTMS augmentation for the treatment of spider phobia. Most subjects were able to complete the combined iTBS and exposure treatment paradigm, and both groups of subjects experienced a significant reduction in subjective and physiological exposure-related distress. Subjects experienced greater discomfort related to stimulation at the vmPFC site as indicated by the lower average intensity of iTBS administered to this location, raising some concerns regarding the tolerability of treatment. At the same time, there were no differences in drop-out rates and we anticipate subjects would adjust to treatment-associated-discomfort with additional treatment sessions (71, 72). Although there were no significant differences in the benefit of the intervention between treatment groups, *post-hoc* analyses revealed that those subjects who received more intense active stimulation experienced a greater reduction in both subjective and physiologic distress with treatment. These analyses, though limited in size and scope, indicate the potential presence of intensity-dependent effects of iTBS in the treatment of phobias.

The strong correlation between the treatment intensity tolerated and multiple physiologic and subjective distress measures during exposure suggests that subjects who were able to tolerate greater stimulation intensity were likely to receive the greatest benefit from iTBS treatment. This finding is consistent with the hypothesis that vmPFC is a promising treatment target for specific phobia and supports the need for further research into vmPFC stimulation, including a more prolonged course of treatment (35). Alternatively, is possible that the ability to tolerate a higher treatment intensity reflects an ability to tolerate increased distress from other sources, meaning that the intensity tolerated and the correlated measures of distress simply reflect one’s distress tolerance. This explanation of effects was not tested in the present study and warrants further research.

Prior work theorizes that network-priming effects during exposure therapy are related to enhancement of the response to exposure rather than an independent effect on extinction recall (33, 35, 60). rTMS has demonstrated both some degree of immediate augmentation of the exposure response and its ability to affect (at least transiently) the networks involved with threat processing (the same networks primed by exposures, as performed in this study) (21, 31, 33, 35, 41, 60, 73). However, rTMS is generally performed without any additional stimulus provided during treatment (i.e., rTMS and exposure therapy are generally performed separately). The effects seen with short courses of combined exposure and rTMS therapy have not yet been studied with longer courses of treatment, and the number of treatment sessions needed to achieve maximal benefit should be examined in future work. Although the mechanism of augmentation is not certain, future research should evaluate whether a longer treatment course may yield additive benefits over time and magnify clinical improvement.

The results of the present study should be interpreted in the context of several limitations. In particular, this proof-of-concept study utilized a small sample size. This substantially limits the power of this study, such that conclusions drawn here are preliminary and should be explored in future work. In addition, this study utilized a homogeneous study population (due to recruitment methods), yielding a primarily college-age population, which may limit the generalizability of these results. Future studies should examine these methods in larger, more heterogenous samples with the goal of determining the efficacy of rTMS as a treatment for phobias. This study also utilized very brief treatment compared both to conventional rTMS and exposure treatment standards (48, 74). It is conceivable that differences between the treatment groups could have been amplified by a greater number of exposure practices, possibly resulting in more subjects completing the BAT after treatment completion, as well as a greater number of TMS treatments, which might have resulted in a larger effect. A more prolonged course of treatment would also have the advantage of allowing the subjects greater time to accommodate to the discomfort of stimulation. Future studies also should employ strategies such as ramping up of intensity in order to improve tolerability of stimulation. Furthermore, although we selected our control condition with the goal of imitating the sensation of rTMS without involving the circuitry implicated in the phobic response (as discussed earlier, and as prior work has done), it is possible that our control condition was not inert (23, 62–65). Additionally, this study involved a substantial average delay between exposures and rTMS. This may have limited efficacy of the rTMS, as prior work has utilized shorter time periods between the two (33, 35, 60). Finally, we noted a large (though not statistically significant) difference in pre-treatment baseline SCL between groups. However, our analyses do not presently indicate that this difference limited our ability to detect physiologic differences between groups.

## Conclusion

This study demonstrates the feasibility of adjunctive use of rTMS with exposure therapy in a sample of phobic subjects. We found improvement across subjects in active and control conditions, and *post-hoc* analyses showed that higher vmPFC rTMS intensity correlated with greater improvements in behavioral and physiologic distress. These data, in conjunction with prior work, preliminarily suggest that rTMS may have beneficial effects to enhance exposure therapy. Further work is needed to explore the beneficial nature of rTMS and to further clarify the protocols and parameters to be used for treatment.

## Data Availability Statement

The datasets presented in this article are not readily available due to privacy or ethical restrictions. Requests to access the datasets should be directed to ML, mkleuchter@mednet.ucla.edu.

## Ethics Statement

The studies involving human participants were reviewed and approved by the UCLA Medical IRB #3. The patients/participants provided their written informed consent to participate in this study.

## Author Contributions

ML, BR, AL, AM, MC, and MI designed the study with input from DK, NG, JCL, SW, RT, JL, and KM. ML, BR, and GS recruited the subjects for the study. BR, GS, and NW performed the behavioral testing and exposure trials and gathered the corresponding data. AL, DK, NG, JCL, SW, RT, JL, and KM performed the TMS treatments. ML and BR analyzed the study data with input and statistical support from AM, AL, MC, and MI. ML and BR primarily drafted the manuscript. All authors contributed to the data interpretation, assisted in manuscript editing, and approved the final manuscript.

## Conflict of Interest

ML discloses that he has served as a consultant to Neuroelectrics, Inc., within the past 36 months. AL has served as a consultant to NeoSync, Inc., Ionis Pharmaceuticals, Inc., and ElMindA. AL is Chief Scientific Officer of Brain Biomarker Analytics LLC (BBA), and has equity interest in BBA. The remaining authors declare that the research was conducted in the absence of any commercial or financial relationships that could be construed as a potential conflict of interest.

## Publisher’s Note

All claims expressed in this article are solely those of the authors and do not necessarily represent those of their affiliated organizations, or those of the publisher, the editors and the reviewers. Any product that may be evaluated in this article, or claim that may be made by its manufacturer, is not guaranteed or endorsed by the publisher.

## Abbreviations

rTMS: repetitive transcranial magnetic stimulation
vmPFC: ventromedial prefrontal cortex
rTMS: repetitive transcranial magnetic stimulation
iTBS: intermittent theta burst stimulation
SPQ: spider phobia questionnaire
FSQ: fear of spiders questionnaire
BAT: behavioral avoidance test
SCL: skin conductance level
PHQ9: patient health questionnaire-9
GAD-7: generalized anxiety disorder-7
HAMD: Hamilton Depression Rating Scale
ET: exposure therapy
MDD: major depressive disorder
GAD: generalized anxiety disorder
RMT: resting motor threshold
AntD: anticipatory distress
MaxD: maximum distress
MLM: multi-level mixed-effects model.
